# The Response of *Enterococcus faecalis* V583 to Chloramphenicol Treatment

**DOI:** 10.1155/2010/483048

**Published:** 2010-06-15

**Authors:** Ågot Aakra, Heidi Vebø, Ulf Indahl, Lars Snipen, Øystein Gjerstad, Merete Lunde, Ingolf F. Nes

**Affiliations:** ^1^Department of Chemistry, Biotechnology and Food Science, The Norwegian University of Life Sciences, P.O. Box 5003, 1432 Ås, Norway; ^2^Department of Mathematical Sciences and Technology, The Norwegian University of Life Sciences, P.O. Box 5003, 1432 Ås, Norway; ^3^Lerum Konserves AS, P.O. Box 159, 6851 Sogndal, Norway

## Abstract

Many *Enterococcus faecalis* strains display tolerance or resistance to many antibiotics, but genes that contribute to the resistance cannot be specified. The multiresistant *E. faecalis* V583, for which the complete genome sequence is available, survives and grows in media containing relatively high levels of chloramphenicol. No specific genes coding for chloramphenicol resistance has been recognized in V583. We used microarrays to identify genes and mechanisms behind the tolerance to chloramphenicol in V583, by comparison of cells treated with subinhibitory concentrations of chloramphenicol and untreated V583 cells. During a time course experiment, more than 600 genes were significantly differentially transcribed. Since chloramphenicol affects protein synthesis in bacteria, many genes involved in protein synthesis, for example, genes for ribosomal proteins, were induced. Genes involved in amino acid biosynthesis, for example, genes for tRNA synthetases and energy metabolism were downregulated, mainly. Among the upregulated genes were EF1732 and EF1733, which code for potential chloramphenicol transporters. Efflux of drug out of the cells may be one mechanism used by V583 to overcome the effect of chloramphenicol.

## 1. Introduction

Chloramphenicol (Cm) has been used as an broad-spectrum antibiotic in human and veterinary medicine since the 1950s, but the use of chloramphenicol in humans is now rather limited [1]. In animals, chloramphenicol use is limited to pets and non-food-producing animals. The structure of chloramphenicol is relatively simple, and it was the first chemically synthesized antibiotic on the market [1].Chloramphenicol inhibits translation in bacteria, by inhibition of the peptidyl transferase reaction of the large subunit of the ribosome. The inhibition of the peptidyl transferase activity is mediated by binding to several proteins in the 50S ribosomal subunit [2]. 

A number of resistance mechanisms to chloramphenicol in bacteria has been described, of which the most common is enzymatic inactivation by acetylation of chloramphenicol via chloramphenicol transferases (CATs) [1]; Chloramphenicol acetyltransferases (CATs) have been described in both Gram-positive and Gram-negative bacteria. CATs catalyze the hydroxylation of chloramphenicol, thereby leaving the antibiotic inactive [1]. The inactivation of chloramphenicol can also be performed by xenobiotic acetyltransferases [1, 3]. A third mechanism of chloramphenicol inactivation is performed by chloramphenicol phosphotransferases [1]. Several examples of chloramphenicol resistance or lowered sensitivity to chloramphenicol due to efflux pumps (specific or multidrug transporters) have been described, mainly in Gram-negative bacteria [1]. Genes encoding CATs and chloramphenicol efflux pumps are regulated by translation attenuation [4]. Finally, chloramphenicol resistance may be due to mutations in 23S rRNA, thereby changing the binding site of chloramphenicol in the cells [1]. 

In this paper we present the transcriptional profile of *Enterococcus faecalis *V583 (V583) treated with chloramphenicol. Enterococci are known to be inherently resistant to many antibiotics, while acquired resistance to several other antibiotics has been described (see e.g., review by Franz et al. [5]). These days, *E. faecalis *is recognized as a notorious opportunistic pathogen, which frequently acquires antibiotic resistance determinants and potential virulence factors. V583 is a multiresistant isolate, but no specific chloramphenicol resistance genes have been identified in its genome sequence. V583 survives treatment with chloramphenicol, but the growth is decreased, which shows that chloramphenicol induces stress on the cells. 

We used DNA microarrays to obtain a profile of the transcriptional events in chloramphenicol treated V583 cells, to identify mechanisms/genes behind the tolerance to chloramphenicol. In this paper we have, in addition to “traditional” analysis of microarray images, used a prototype method for automated analysis of microarray images, which reduce the manual interference with image analysis. The use of DNA microarrays to monitor transcription in bacteria is very useful, since it may help to identify specific as well as general mechanisms of stress tolerance/adaptation. It also adds crucial information to the growing amount of genomic data on bacterial behavior.

## 2. Materials and Methods

### 2.1. Bacterial Strain, Growth Conditions, and RNA Isolation

*E. faecalis *V583 was grown aerobically overnight in flasks with GM17 medium (Difco) at 37°C on a rotary shaker (300 rpm). To determine the level of chloramphenicol to be used in cultures prior to the microarray experiments, cells were grown in GM17 medium containing 10, 7.5, 5.0, 2.5, and 0 *μ*g/ml chloramphenicol. Growth was monitored for 4 hours. For the transcription analyses (microarray experiments) V583 cells were treated with 2.5 *μ*g/ml chloramphenicol.

For the microarray experiments, cultures grown overnight were diluted 50× and grown in GM17 as above, for approximately 1 hour, to OD_600_ ~0.2. Then, cultures were split in two, and chloramphenicol (Sigma) was added to one of the cultures. The final concentration of chloramphenicol was 2.5 *μ*g/ml. The two cultures (GM17 and GM17 plus chloramphenicol) were then incubated further and 5 ml samples of each culture were collected at 0 (t0), 90 (t90), and 180 (t180) min after addition of chloramphenicol. OD_600 _was measured for all samples. Cell harvesting, RNA extraction, determination of integrity, and concentration of RNA were performed as described previously [6].

### 2.2. Transcription Profiling with the Use of DNA Microarrays

The microarrays used in this work have been described previously by Aakra et al. [6], cDNA synthesis, fluorescent labeling, hybridization, and image acquisition were performed as described previously [6]. RNA was extracted independently from three experiments. At least five replicate hybridizations were performed for each time point. Microarray experiments were performed as dye-swap experiments, to avoid bias introduced by differential labeling of the cDNA.

### 2.3. Image Analysis

The microarray images were analyzed twice; with the GenePix Pro 6.0 (see [7]) and with a prototype program for microarray image analysis (see [8, 9]).

### 2.4. The Development of a Prototype Program for Analysis and Quality Assessment of Microarray Images

A prototype program competitive to established methodology on DNA microarray analysis was used [8, 9]. Central aspects in development of this program were (1) spot segmentation, (2) intensity modeling and calculation of gene expression values, and (3) quality assessment of the spots on the microarray images. 

For each spot box (grid cell) the solution of (1) included estimation of bivariate Gaussians, rearrangement of the pixels into a one-dimensional sequence defined by the Gaussian and solution of the spot segmentation problem (identification of the foreground spot pixels) by maximizing associated two-sample *t*-statistics [8, 9]. The solution of (2) used the rearranged sequence of pixels as a basis for logistic modeling to estimate fore- and background levels (Cy3- and Cy5 channels were considered separately). Traditional calculations of associated log-ratios were then based on these estimated fore- and background levels. Quality assessment (3) was based on a set of decision rules, which involved 12 distinct quality parameters defined in close association with the estimated Gaussians (i.e., shape, size, and position of the spots) and the logistic models, that is, features of the fitted curves and model residuals [8, 9].

### 2.5. Downstream Analysis of Data

Downstream analysis was done by the LIMMA package (www.bioconductor.org) in the R computing environment (www.r-project.org). Preprocessing and normalization followed a standard procedure using methods described by Smyth and Speed [10]. Testing for differentially expressed genes was done in a linear model context as described by Smyth [11]. A mixed-model approach was chosen to describe within-array variation between replicates (5 replicates of each probe in each array), and an empirical Bayes smoothing of gene-wise variances was conducted according to Smyth et al. [12]. For each gene, *P*-values were adjusted to control the false discovery rate, hence all *P*-values displayed are adjusted for false discovery rates (FDR-adjusted; often referred to as *Q*-values in the literature).

### 2.6. Real-Time RT-PCR

To confirm independently the differential gene expression observed by microarray experiments, the following genes were selected for analysis by real-time quantitative reverse transcription PCR (RTQ): EF0633 (*tryS-1, *encoding tyrosyl-tRNA synthetases), EF2653 (encoding a transcriptional regulator of the Cro/CI family), and EF0105 (*argF-1*, encoding ornithine carbamoyltransferase). EF1964 (*gap-2, *encoding glyceraldehydes-3-phosphate dehydrogenase), which is constitutively expressed, was used to normalize the *Taq*Man data. The RTQ analyses were run as described previously by Aakra et al. [6].

### 2.7. Microarray Accession Number

The microarray data have been deposited in the ArrayExpress database (http://www.ebi.ac.uk/arrayexpress/) with the series accession number (not yet available).

## 3. Results

### 3.1. Growth of *E. faecalis* V583 in Presence of Chloramphenicol

**Figure 1:**
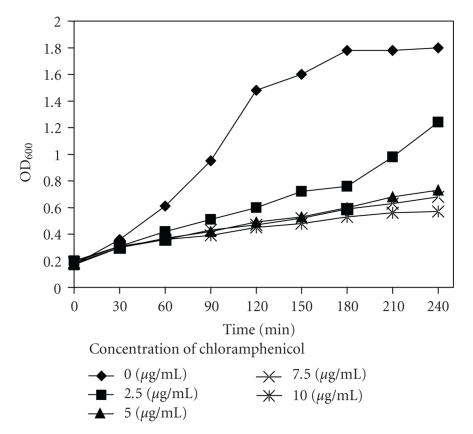
Growth of *E. faecalis* V583 treated with chloramphenicol, measured spectrophotometrically (600 nm). Cell samples used for the microarray hybridizations were collected immediately after addition of chloramphenicol (2.5 *μ*g/*μ*l), after 90 min and after 180 min.

To determine suitable concentrations of chloramphenicol in the transcription profiling experiments, V583 was treated with various concentrations (2.5 *μ*g–10 *μ*g per ml) of chloramphenicol. Growth of V583 decreased at all concentrations of chloramphenicol. After 90 min OD_600_ of all chloramphenicol-treated cultures was <50% of the OD_600_ of the untreated control (Figure 1). For the microarray experiments, it was decided to treat the V583 cells with 2.5 *μ*g/ml chloramphenicol. For transcriptional profiling experiments using microarrays, it has been recommended to use low inhibitor concentrations, since higher concentrations may induce secondary responses [13, 14].

### 3.2. Transcription Profiling of Chloramphenicol-Treated V583 by Microarrays

The microarray experiments were performed as time course experiments, where cell samples for RNA extraction and further hybridizations were collected three times after addition of chloramphenicol to one of the V583 cultures. Untreated cells were used as controls in all experiments. Cell samples were collected immediately after addition of chloramphenicol (t0), after 90 min (t90) and after 180 min (t180). Corresponding cDNA samples for each time point were mixed and hybridized to microarray slides. 

For determination of differential transcription, genes were “scored” as significantly differentially transcribed, with threshold *P* < .05. In the present context, where results from the use of two different methods for image analysis were to be compared, it was useful to have a consistent method to determine whether each gene was differentially transcribed or not. When the transcription of genes was higher in the presence of chloramphenicol, the gene expression is denoted upregulated (induced), and when the transcription was lower, the gene expression was denoted downregulated (repressed).

### 3.3. Analysis and Quality Assessment of Microarray Images

We applied two different methods for analysis of the microarray images obtained during the experiments: The GenePix software and a prototype program. The prototype microarray image analysis method analyses the spots on the images automatically, based on numerous quality parameters, which reduces the manual interference with the analysis considerably. Most genes that were scored as significantly differentially expressed using GenePix were also found significantly differentially expressed by the prototype program, and vice versa; 609 genes were scored significantly up- or downregulated at one or more time points both in the data based on GenePix and the prototype analyses. Of these, 23 were plasmid encoded. By the use of the prototype program and *P* < .05 as a threshold for significance, 694 chromosomal (27 plasmid) genes were scored as significantly up- or downregulated at one or more of the three time points. Contrary, based on the GenePix data, 672 (28 plasmid) genes were scored as significantly differentially expressed. Those genes that were scored as significantly up- or downregulated by only one of the methods were mainly represented on the microarrays by spots with weak signal intensities. The *P*-value associated with the expression data of these genes were, for most genes, close to the threshold value for significance (*P* > .05). 

We decided to go on with the genes (609) that were scored as significantly differentially expressed in both datasets. The transcriptional patterns of plasmid genes (pTEF1, pTEF2, and pTEF3) are dealt with in a separate section (see below).

Chloramphenicol treatment appeared to have a significant effect on transcriptional events in the V583 cells, as seen by the high number (609) of genes that were differentially transcribed at one or more time points. 301 genes were downregulated, 336 genes were upregulated at one or more time points. Common patterns of regulation were seen; 15 genes were differentially transcribed at all three time points, while 121 genes were differentially transcribed at two time points. The number of differentially transcribed genes was highest at t90 (lowest at t0). The differential transcription is probably due to (1) the chloramphenicol treatment and (2) differences in growth rate between the treated and untreated cultures. For 25 genes we found that the transcription level varied (up and down) during the time course. 

Among the genes with altered transcription, at one or more time points, genes encoding hypothetical proteins constituted the largest group, but these genes also constitute the largest group in the V583 genome. Relative to the number of genes encoding hypothetical proteins among the V583 genes (>1/3), the number of differentially transcribed genes (both upregulated and downregulated) belonging to this group is rather low (data not shown). Genes related to transport and binding processes also constitute a large group among the differentially transcribed genes, but the number of downregulated genes in this category is higher than the number of upregulated genes: The genes encoding transport and binding proteins constitute nearly 12% of the V583 genes, 12% of the downregulated genes, and 6% of the upregulated genes. Among the upregulated genes, were genes belonging to the categories protein synthesis; purines, pyrimidines, nucleosides, and nucleotides; as well as fatty acid and phospholipid metabolism were enriched. Among the downregulated genes were amino acid biosynthesis genes, and energy metabolism genes. 

As mentioned briefly above, 15 genes showed significant expression at all three time points examined (see Table 1). Most of these genes (12) were upregulated at all time points. The cystathionine beta-lyase gene (EF0290) was downregulated at t0 and t90 and upregulated at t180. The chaperonin encoding genes (EF2633 and EF2634, *groE* operon) were downregulated at t0 and t180 and upregulated at t90. This operon (*groE*) was the only operon, in which genes were regulated at all points during the time course. 

An important characteristic of the V583 genome sequence is the high amount of probable mobile genetic elements and exogenously acquired DNA (MGE) [15]. These genes constitute about 1/4 of the V583 genome sequence; 54 of the MGE genes (out of approx 680 chromosomal MGE genes) were differentially transcribed. For example, 13 genes belonging to the phage01 (51 genes total. See [16] for designation) were upregulated in the chloramphenicol-treated cells. Among these was the endolysin-encoding gene (EF0355). Genes from the phage05, phage06, and phage07 were also differentially transcribed; the phage05 genes were downregulated, while the phage06 and phage07 genes were upregulated (except for EF2822, which was downregulated at t0). Another endolysin-coding gene (EF2802) is located in the phage06 region, and this gene was among the upregulated phage06 genes. In the phage02 (EF1276–EF1293) no genes were differentially transcribed. The phage02 is found in many different *E. faecalis *strains [16–18]. Several genes from the pathogenicity island (PAI; see [15, 19]) and from the region comprising the *vanB* resistance determinants (see [15]) were differentially transcribed. The PAI genes were mainly repressed (7 out of 9), while most of the differentially transcribed genes from the* vanB *region were induced (8 out of 11). Among the induced *vanB *region genes were the genes encoding the histidine kinase VanSB and its cognate response regulator, VanRB. 

To be better able to determine which genes were more important in the response of V583 to chloramphenicol treatment, we examined differentially transcribed operons (see http://www.microbesonline.org/operons/gnc226185 .html and [20]) with differential transcription. In the operon EF0205–EF0234, encoding ribosomal proteins (r-proteins), eight genes were upregulated. Other putative operons encoding ribosomal proteins were also upregulated; EF0915–EF0916, and EF2715–EF2716. Among the differentially transcribed genes, which encode r-proteins, only one (EF0820) was repressed. 

In the operon containing genes for V-type ATPase (EF1492–EF1500), four genes were upregulated. The upregulation of genes in this operon was also seen in the study by Solheim et al. [7] in V583 treated with bovine bile and in V583 treated with bovine bile and SDS. Another induced operon was the *pyr* operon (EF1712–EF1721), which was induced in V583 treated with bovine bile as well [7]. Most of the genes in the operon EF2875–EF2885, which encodes genes involved in fatty acid biosynthesis, were induced (t90 and t180). The EF1732 and EF1733 genes encode two potential transporters of chloramphenicol out of the V583 cells; these two genes were both strongly induced in the V583 cells (t90).

### 3.4. Differential Transcription of Genes on the pTEF Plasmids

The three plasmids of the V583 genome are all similar to well-known plasmids [15]. Antibiotic resistance genes (e.g.,* ermB*) and some putative virulence factors (e.g., aggregation substance) are among the genes that are specified by the plasmids [15]. In the chloramphenicol-treated V583 cells, only a few of the plasmid encoded genes were differentially transcribed: eleven genes (one up, ten down regulated) from pTEF1, eight genes (three upregulated, five/5 downregulated) from pTEF2 and four genes from pTEF3 (one downregulated, three upregulated) were differentially transcribed. Among the differentially transcribed (downregulated) plasmid genes was one gene coding for drug resistance or transport (EFA0010).

## 4. Discussion

In this paper, we report the transcriptional patterns of *E. faecalis *V583 treated with chloramphenicol. *E. faecalis* V583 is able to grow in media containing chloramphenicol, but compared to optimal growth, it then grows slowly. No specific genes coding for chloramphenicol resistance have been identified in V583, and the ability to grow in the presence of chloramphenicol is not due to mutations in the 23S rRNA gene. To study the transcriptional patterns of V583 cells treated with chloramphenicol, a time course experiment was chosen, since such experiments might provide clues on how the bacterium adapts to the drug. We used two different methods for image analysis prior to estimation of transcription levels, and results were consistent. For example, the differential regulation of numerous operons corroborates the consistency and reliability of the results from the microarray experiments. Moreover, the number of induced and repressed genes was similar, as expected in experiments like this. The transcription patterns were also consistent with those of other similar studies. For example, genes encoding ribosomal proteins were upregulated, and genes for tRNA synthetases were downregulated. Genes involved in fatty acid biosynthesis were also induced, as well as genes coding for a V-type ATPase, which was seen also in another study of stress response in V583 [7]. The induction of the genes for the V-type ATPase points to the importance of optimal redox conditions in the stressed (treated) cultures, as discussed also by Solheim et al. [7].

**Functional Groups**

For annotation of genome sequences the classification of genes into functional groups, is useful. The largest group of genes in V583 (and most other genomes) are genes encoding hypothetical proteins and conserved hypothetical proteins. They constitute more than 1/3 of the genome, and, not surprising, these genes are a major group of regulated genes in transcription profiles [6, 7, 21, 22]. The importance of the genes for hypothetical proteins has been discussed in many papers, see for example [23–25]. In our work, we found that the genes encoding hypothetical proteins actually constituted a smaller group of the regulated genes than what was expected based on the pure number of these genes in the genome sequence. Genes specifying proteins involved in protein synthesis, fatty acid, and phospholipid metabolism were enriched among the regulated genes, compared to their fraction in the genome sequence. Although the functional classes of genes are defined broadly, this may reflect which genes and groups are specifically important in the response to chloramphenicol treatment in V583.

The pyrimidine biosynthesis operon (*pyr*; EF1721–EF1712) is regulated by transcriptional attenuation, where *pyrR* (EF1721, first gene in the operon) encodes the regulator causing transcription termination [26]. Some bacteria must be able to perform pyrimidine synthesis to be virulent [27]. Carbamoyl phosphate is the precursor for pyrimidine biosynthesis and for arginine biosynthesis. In the chloramphenicol-treated V583 cells, *pyr *was upregulated (t180, mainly), while genes specifying arginine biosynthesis were downregulated (t90 and t180). Differential transcription of these genes has been observed also in previous papers [6, 7]. Since the complete *pyr *operon was upregulated in the chloramphenicol-treated V583 cells, we may assume that this is related specifically to the chloramphenicol addition in the cultures.

Genes involved in fatty acid biosynthesis (e.g., EF0282–EF0284 and EF2875–EF2885) were enriched among the induced genes (t180, mainly). Some of these genes were induced also in V583 cells treated with SDS [7]. The induction of these genes points to alteration of the cell membrane as one mechanism for adaptation of V583 to stress during time courses. An altered membrane may change the transport capabilities of the cell, which may partly explain the relatively low number of induced genes specifying transport and binding proteins (low fraction compared to the size of this group in the genome).

**MGE Genes**

Compared to previously published transcription profiles of V583 [6, 7], we observed that a substantial amount of the exogenously acquired genes of V583 was expressed in this study. The induction of phage genes (e.g., genes coding for endolysin) may be an indication of a certain extent of lysis of the chloramphenicol-treated cells.

**Differential Transcription of Genes Involved in Protein Synthesis**

Since rRNA is the cellular target of chloramphenicol, a transcriptional response of genes encoding ribosomal proteins (r-proteins) was expected. Indeed, several genes coding for r-proteins were induced, and the overproduction of these proteins may partly explain the tolerance of V583 to chloramphenicol. However, the synthesis of many r-proteins is regulated at the translational level, and, therefore microarray analyses may not reflect the actual level of r-proteins in the chloramphenicol treated cells.

Genes coding for aminoacyl tRNA synthetases were downregulated (t90), while the prolyl tRNA synthetase gene was upregulated. The repression of aminoacyl tRNA synthetase genes was seen also in the study by Ng et al. [28], where the transcription patterns of *Streptococcus pneumoniae *treated with different translation inhibitors were described. The repression of the tRNA synthetase genes may express lower rate of protein synthesis in the treated cells. The untreated cells, which grew faster, need to synthesize proteins faster than the untreated cells. The repression of the tRNA synthetase genes, may, though, appear peculiar, since genes for other parts of the translation apparatus (genes encoding ribosomal proteins and elongation factors) were induced. Antitermination regulates expression of most aminoacyl tRNA synthetases and other enzymes involved in amino acid biosynthesis (review by Ryckelynck et al. [29]).

**Transcription of Genes for Drug Transport**

No chloramphenicol resistance genes have been identified in the V583 genome. In the V583 genome sequence, there are several genes coding for putative drug transporters. One of these genes were down regulated, (EFA0010). The EF1732 and EF1733 genes, however, which encode ABC transporters in the MDR family, were upregulated (t90 (EF1732); all time points (EF1733)). These two genes were induced in V583 cells treated with erythromycin, also [6], which points to them as potentially important drug transporters in V583. They are, thus, obvious targets for future detailed experiments, like knockout studies. Genes corresponding to EF1732 and EF1733 appear widespread in *E. faecalis* [18], and their function in other strains than V583 should also be further elucidated. Efflux of chloramphenicol appears as one of the mechanisms that may confer the resistance in V583. V583 cells treated with chloramphenicol grow very slowly immediately after addition of the drug, while the growth rate increases later (Figure 1). The changed growth rate supports the hypothesis on efflux of chloramphenicol as one resistance mechanism.

Chloramphenicol binds to the 23S rRNA by hydrogen bonding [2], and probably, an equilibrium between bound and unbound chloramphenicol in the cells are established. The efflux of free-drug molecules will then lead to release of bound chloramphenicol from the ribosome.

## Figures and Tables

**Table 1:** Genes whose expression was significantly different from 0 (upregulated or downregulated during the whole time series (three time points)).

		*t0*		*t90*		*t180*	
Gene	Function	logFC ML^1^	logFC GP^2^	logFC ML	logFC GP	logFC ML	logFC GP
EF0290	Cystathionine beta-lyase	−0.45	−0.46	−1.66	−1.36	1.07	0.97
EF0727	Conserved hypothetical protein TIGR00147	0.65	0.64	0.40	0.38	1.42	1.34
EF1527	P-binding protein	0.82	0.83	0.56	0.57	1.31	1.28
EF1694	Ribosomal protein S16	1.25	1.28	0.91	0.95	1.62	1.74
EF1733	ABC transporter	0.48	0.51	3.00	2.96	2.77	1.79
EF2173	Transposase	0.62	0.61	0.43	0.48	1.12	1.36
EF2185	Transposase	0.73	0.70	0.89	0.84	1.08	1.15
EF2420	Homoserine kinase	0.63	0.55	0.71	0.69	1.28	1.28
EF2443	Ribosomal protein S20	0.81	0.76	0.80	0.81	1.83	1.84
EF2633	Chaperonin	−0.56	−0.52	0.89	0.89	−2.79	−2.67
EF2634	Chaperonin	−0.87	−0.90	0.99	1.00	−2.11	−1.83
EF2868	Conserved hypothetical protein	0.57	0.54	1.00	1.00	1.71	1.49
EF2973	Alkaline phosphatase	0.72	0.64	1.46	1.25	1.23	1.14
EF3254	1,4-dihydroxy-2-naphthoate octaprenyl transferase, putative	0.65	0.66	1.45	1.32	1.58	1.43
EF3295	Hypothetical protein	0.51	0.49	0.49	0.49	2.09	1.90

^1^logFC ML; log-value of transcription ratio of gene based on the analysis using the prototype image analysis program;

^2^logFC GP; log-value of transcription ratio of gene based on the analysis using the GenePix method for image analysis.
